# Factors Associated with the Use of Complementary and Alternative Medicine/Therapy among United States Adults with Asthma

**DOI:** 10.3390/healthcare11070983

**Published:** 2023-03-30

**Authors:** Chukwuemeka E. Ogbu, Chisa O. Oparanma, Russell S. Kirby

**Affiliations:** 1 Chiles Center, College of Public Health, University of South Florida, Tampa, FL 33612, USA; 2 Department of Medicine, Kharkiv National Medical University, 61022 Kharkiv, Ukraine

**Keywords:** complementary and alternative medicine, asthma, asthma control, United States

## Abstract

This article examined the sociodemographic and health-related factors associated with the use of complementary and alternative medicine/therapy (CAM) among adults with current asthma in the United States. We used data from 76,802 adults aged 18 years and above from the 2012–2019 Behavioral Risk Factor Surveillance System (BRFSS) Asthma Call-back Survey (ACBS) cycles. Weighted binary and multinomial logistic regression was used to examine the association of these factors with ever CAM use and the number of CAM use. We found that approximately 45.2% of US adults with asthma ever used CAM. Among adults with asthma, 25.3% and 19.9% endorsed using one CAM and ≥2 CAMs, respectively. CAM use was significantly associated with adults ≤ 35 years, female gender, multiple/other race/ethnicity, higher cost barriers, adults with two or more disease comorbidities, and those with poorly controlled asthma in both binary and multinomial models. CAM use was not associated with insurance and income status. Understanding factors associated with CAM use can provide asthma care professionals valuable insights into the underlying drivers of CAM use behavior in this population, enabling them to offer more informed and effective medical advice and guidance.

## 1. Introduction

Asthma is a chronic inflammatory lung condition affecting 21 million adults in the United States (US) [1]. Conventional asthma therapy includes the use of control agents, such as inhaled corticosteroids, bronchodilators, such as beta-agonists and anticholinergics, theophylline, leukotriene modifiers, and more recently, the use of anti-immunoglobulin E (IgE) antibodies, anti–IL5 antibodies, and anti–IL4/IL13 antibodies in selected patients [2]. These medications work by reducing the bronchoconstriction and airway inflammation associated with the disease and significantly reduce asthma disease burden. Even with the presence of these medications, there has been an increasing prevalence in the use of complementary and alternative medicine (CAM) [3]. The National Center for Complementary and Integrative Health defines complementary and alternative medicine (CAM) as “a group of diverse medical and health care systems, practices, and products that are not generally considered part of conventional medicine” [4]. In essence, if a person with asthma is treated outside of the earlier described conventional therapy, they are said to be using complementary or alternative medicine. When used together with conventional medicine, it is considered complementary, and when it is used instead of conventional medicine, it is regarded as an alternative [4]. A nationwide survey in 2012 estimated that more than 30% of people in the United States are using complementary or alternative medicine [4], and an estimated 47.6% are doing so without the knowledge of their physician [5].

Patients with asthma may opt to use complementary and alternative medicine (CAM) for a range of reasons, including a preference for natural remedies over synthetic drugs [6], dissatisfaction with conventional medicine due to inadequate symptom relief or medication side effects [7], a desire for a holistic approach [8], and limited access to conventional medical facilities and care during times of need [9]. In healthcare settings, factors, such as physician burnout, anxiety, depression, and a perceived lack of caregiver empathy, may weaken the patient–physician relationship and motivate individuals to pursue alternative treatment options [10,11]. Additionally, the high financial costs associated with conventional asthma management [12,13,14] may prompt patients to explore more affordable therapies, such as breathing techniques or acupuncture [14], although some CAM treatments, such as aromatherapy, may themselves be expensive due to the need for specialized equipment or lengthy treatment periods [15]. The availability of insurance and the individual’s income level can also act together to influence the likelihood of CAM use [16,17,18]. Finally, some patients may turn to CAM because these therapies align with their cultural or religious traditions [19].

Among the numerous available complementary and alternative medicines, the most commonly used for asthma include breathing exercises, non-vitamin, non-mineral dietary supplements (i.e., herbs), and vitamins [16]. Research evaluating the efficacy of CAM shows little evidence of clinical benefit, possibly due to poor methodology and methodological differences among studies. A study by Santino et al. (2020) determined that among patients who used breathing exercises, the quality of evidence for the measured outcomes ranged from moderate to very low certainty according to GRADE criteria [20]. Other papers suggest that the safety and functional mechanism of the ingredients in herbal medicine is inconclusive and must be further evaluated [21,22].

Studies exploring the factors associated with CAM use among US adults with asthma are limited and understanding these factors can help healthcare providers offer informed medical advice to patients and enable health policy professionals to understand the cost and health resource factors linked to CAM use among adults in this population. Prior research examining the characteristics and health perceptions of CAM use among the general US adult population identified age, sex, race, and income as factors associated with CAM use [5]. Studies among asthma patients have shown that middle-aged adults (aged 45 to 65) are more likely to use alternative treatments [3,23]. At the same time, persons under 18 are less likely to use CAM, as concluded in a study by Shen et al. (2011) [24]. Several studies have reported a higher prevalence of CAM use among women and the likelihood of CAM use among the female gender [23,25,26,27]. Caucasian adults and American Indian or Alaska Native adults were more likely to use CAM than Asian adults or African American adults [26,28]. Some studies have suggested that asthma control patterns and asthma severity are directly associated with CAM use [16,29,30,31,32,33]. However, the small sample size, the inability to extrapolate findings to the US adult asthma population [16], and the lack of control of baseline comorbidities [16,31] associated with CAM use were some study limitations.

To gain insight into the determinants of CAM use, we have applied Andersen’s Behavioral Model of Healthcare Service Use [34]. This model is a comprehensive, multilevel framework that incorporates both individual and contextual determinants of healthcare service utilization [34]. In this model, factors associated with healthcare use are broadly categorized into three major components: predisposing factors that are exogenous to the healthcare system (e.g., demographic characteristics, such as age, gender, race/ethnicity, social relationships, education, and income), enabling factors that are necessary but insufficient for service utilization (e.g., health insurance and healthcare cost barriers), and need-based factors that are related to the perceived need for health services and a patient’s health status (e.g., asthma control status and comorbidity status) [34,35]. Therefore, our study aims to evaluate these factors associated with CAM use among US adults with asthma. We examine the relationship between socioeconomic factors, sociodemographic characteristics, asthma impairment measures, comorbidity status, and cost barriers and CAM utilization in the United States. Our goal is to provide valuable insights for public health policy and practice, particularly given the upward trend in CAM use among US adults.

## 2. Materials and Methods

### 2.1. Study Population

The 2012 to 2019 Adult Behavioral Risk Factor Surveillance System (BRFSS) Asthma Call-back Survey (ACBS) was used for this study. The BRFSS is a state-based cross-sectional study of non-institutionalized adults, 18 years and above, residing in the United States. Funded by the Centers for Disease Control and Prevention (CDC), the BRFSS collects data on health risk behaviors, health care access, utilization and preventive health practices, as well as infectious disease and chronic diseases. The ACBS is a continuous survey conducted biweekly after the BRFSS and gathers annual data on demographics and illness histories of individuals with asthma. The survey is designed to address crucial questions concerning the health and experiences of people with asthma and offers state and local level data. Eligible participants for the ACBS are BRFSS adult respondents who report an asthma diagnosis. A positive response to the questionnaire items “Have you ever been told by a doctor, nurse, or other health professionals that you have asthma?” and “Do you still have asthma?” were employed to determine lifetime asthma and current asthma status, respectively. Response rates for ACBS were evaluated using the Council of American Survey and Research Organizations guidelines. The ACBS response rates ranged from 36.9% to 93.0% among participating states, colonies, and Washington DC in the 2012 to 2019 survey period [36].

A total of 76,802 adult respondents who reported a current diagnosis of asthma between the years 2012 and 2019 were included in this study. The ACBS obtained institutional review board approval from the relevant states, as well as the Asthma and Community Health Branch of the National Center for Environmental Health’s Ethics Review Board, and informed consent was provided by all participants. Since the ACBS is a public use dataset, this study is exempt from full institutional review board review. Further details regarding the data, sampling method, and analytical guidelines can be found elsewhere and at https://www.cdc.gov/brfss/acbs/index.htm (accessed on 10 January 2023) [37].

### 2.2. Outcome Ascertainment: Complementary and Alternative Medicine/Therapy Use

During the ACBS survey, participants were initially asked if they utilized any complementary medicine or alternative therapies to manage their asthma. To assess the use of non-traditional, complementary, and alternative healthcare or asthma treatments, the survey item “In the past 12 months, have you used…to control asthma?” was employed. Eleven alternative treatments were evaluated in this manner, including herbs, vitamins, acupuncture, acupressure, aromatherapy, homeopathy, reflexology, yoga, breathing techniques, naturopathy, and other therapies. Responses were binary, recorded as either “Yes” or “No”. We created two outcome variables. The first variable, “Ever use of CAM,” was designed to indicate the use of at least one CAM method to control asthma. Participants who recorded a “Yes” response to any of the eleven therapy categories were reported as having ever used CAM to manage their asthma. The second variable, “Number of CAM use,” was generated by summing up the number of “Yes” responses and categorizing the total as none, one CAM use, and two or more CAM use.

### 2.3. Covariates Ascertainment

Asthma control: Three measures of asthma impairment were utilized as proxies for asthma control: daytime symptoms, night-time symptoms, and use of short-acting β2-agonists (SABA) for symptom control (not for prevention of exercise-induced bronchospasm). Following the approach of Zahran et al. (2015), we constructed an asthma control variable comprising three distinct and non-overlapping categories: well-controlled asthma (daytime symptoms ≤2 days a week, night-time awakenings ≤2 times a month, and short-acting β2-agonists used for symptom control ≤2 days a week), not-well-controlled asthma (daytime symptoms >2 days a week, night-time awakenings 1–3 times a week, and short-acting β2-agonists used for symptom control >2 days a week), and very-poorly-controlled asthma (daytime symptoms throughout the day, night-time awakenings ≥4 times a week, and short-acting β2-agonists used for symptom control several times a day) [38]. This adaptation of the 2007 National Asthma Education and Prevention Program Expert Panel Report 3 Guidelines was necessary due to the limited clinical measures available through the ACBS, which precluded the use of all the required clinical measures for current impairment (such as pulmonary function measures) and for future risk assessment (such as asthma exacerbations or progressive decline in lung function in adults) [39]. Other studies that have employed the ACBS to assess asthma control have also adopted the NAEPP guidelines [38,40].

Disease comorbidity status: Disease comorbidity status was evaluated by computing the sum of various diagnoses obtained from the BRFSS questionnaire, which included depressive disorder, heart attack, angina/coronary artery disease, stroke, skin cancer, any other cancer diagnosis, diabetes, COPD, arthritis, kidney disease, and morbid obesity. The comorbidity status was subsequently categorized into five levels, specifically, none, one comorbidity, two comorbidities, three comorbidities, and four or more comorbidities, to ascertain the burden of concurrent diseases in the participants. A prior systematic review has shown that the total number of diseases can predict health outcomes and perform comparably to more complex measures of disease morbidity [41].

Cost barriers: The evaluation of cost barriers to asthma care was performed across three domains in the ACBS survey, encompassing the cost of seeing a primary care physician, the cost of seeing a specialist for asthma care, and the cost of purchasing asthma medication within the past 12 months. By summing the affirmative responses across these domains, a composite categorical variable was derived. We categorized patients into distinct groups based on the number of cost barriers encountered, ranging from none to one, two, and three cost barriers.

Additional covariates in this study include demographic and socioeconomic factors, specifically age stratified as follows: 35 years or less, 36–50 years, 51–65 years, and greater than 65 years of age, gender (male or female), race (White non-Hispanic, Black, Hispanic, multiracial), health insurance coverage (yes or no), and income divided into four categories (greater than $75,000, between $50,000 and $75,000, between $25,000 and $49,999, and less than $25,000).

### 2.4. Statistical Analysis

The ACBS employs a stratified, multistage survey design to improve the representativeness of the US adult population with asthma. Complex analytical procedures recommended by the ACBS were followed, and appropriate weighting was applied to account for the sampling strata, cluster, and primary sampling unit (PSU).

Bivariate analysis between covariates and the dependent variables (ever use of CAM and number of CAM use) was performed using the Rao chi-square test. Covariates were initially identified based on a review of the literature on factors associated with CAM utilization. These explanatory variables were then selected using both forward and backward selection methods.

In the current study, we examined two dependent variables: the dichotomous measure of at least one CAM use (yes vs. no) and the multinomial measure of the number of CAM use, categorized into three levels: no CAM use, one CAM use, and two or more CAM use. We estimated unadjusted and adjusted weighted binary logistic regression models for the first dependent variable and unadjusted and adjusted weighted multinomial logistic regression models for the second dependent variable. Odds ratios (ORs) and corresponding 95% confidence intervals (CIs) were reported for all independent variables in both models. Additionally, we adjusted for baseline smoking and survey year, in addition to all covariates in the model. Weighted percentages were used to report all percentages. We checked for multicollinearity by examining tolerance estimates and the Variance Inflation Factor (VIF) and found no issues as no tolerance estimate was below 0.1, and no VIF was above 10 [42]. The statistical significance of the hypothesis was assessed using a two-sided *p*-value less than 0.05 or non-overlapping 95% CIs. The data analysis was conducted using SAS 9.4 statistical software (SAS Institute, Cary, NC, USA).

## 3. Results

Among 76,802 adult respondents with current asthma in our study sample, 32,455 (45.2%) reported using at least one CAM (Table 1).

The proportion of respondents who reported ever using CAM was significantly greater than the proportion who reported not using CAM among the following groups: adults ≤ 35 (50.9% vs. 49.1%), respondents of multiple/other race ethnicity (51.9% vs. 48.1%), respondents without a health insurance (53.3% vs. 46.7%), and adults who endorsed one cost barrier (57.2% vs. 42.8%), two cost barriers (65.5% vs. 34.5%), and three cost barriers (69.7% vs. 30.3%). The proportion of adults who endorsed ever using CAM compared to never CAM users was significantly greater among those with very poorly controlled asthma symptoms (57.5% vs. 42.5%) and not well controlled asthma symptoms (50.5% vs. 49.5%). Respondents who ever used CAM had a lower proportion of no comorbidity (41.3% vs. 58.7%), one comorbidity (43.7% vs. 56.3%), two comorbidities (45.7% vs. 54.35%), three comorbidities (47.0% vs. 53.0%), and ≥4 comorbidities (49.5% vs. 50.5%) compared to non-CAM users. Compared to the proportion of non-CAM users and single-CAM users, respectively, adults who used ≥2 CAM endorsed having a higher proportion of two cost barriers (34.7% vs. 30.8% vs. 34.5%) and three cost barriers (40.4% vs. 29.2% vs. 30.3%) (Table 2).

In the binary regression model, males where 30% less likely than women to use CAM (adjusted odds ratio (aOR) = 0.70; 95% CI, 0.64–0.76) (Table 3).

Compared to adults ≤35 years, adults 36–50 years (aOR = 0.71, 95% CI, 0.62–0.81), adults 51–65 years (aOR = 0.60, 95% CI, 0.54–0.68), and adults >65 years (aOR = 0.43, 95% CI, 0.38–0.49) were less likely to use CAM. Respondents who indicated “multiple/other” race/ethnicity were 1.37 (95% CI, 1.16–1.63) times more likely than non-Hispanic White respondents to use CAM. Adults with one, two, and three cost barriers were 54%, 97%, and 110% more likely to use CAM, respectively, compared to adults with no cost barriers (aOR = 1.54 (95% CI, 1.36–1.76), aOR = 1.97 (95% CI, 1.56–2.48), (aOR = 2.10 (95% CI, 1.60–2.75)). Having two comorbidities (aOR = 1.17 (95% CI, 1.02–1.35)), three comorbidities (aOR = 1.28 (95% CI, 1.09–1.51)), and ≥4 comorbidities (aOR = 1.42 (95% CI, 1.22–1.66)) were statistically associated with CAM use. In comparison to adults with well-controlled asthma symptoms, those with not well controlled symptoms (aOR = 1.83 (95% CI, 1.66–2.02)) and very poorly controlled symptoms (aOR = 2.34 (95% CI, 2.09–2.62)) had higher odds of endorsing CAM use.

When one and ≥two CAM users were compared to non-CAM users, males were less likely to use one CAM (aOR = 0.74 (95% CI, 0.67–0.82)) and ≥two CAM (aOR = 0.65 (95% CI, 0.57–0.73)) compared to women (Table 4).

Multiple/other non-Hispanic race/ethnicity was associated with one CAM use (aOR = 1.28 (95% CI, 1.03–1.58)) and ≥ two-CAM (aOR = 1.51 (95% CI, 1.24–1.83)). Odds of single-CAM use when compared with non-CAM users was 36% higher in those who had one cost barrier compared to no cost barrier (aOR = 1.36 (95% CI, 1.17–1.57)) and 82% higher in those who had ≥two cost barriers compared to those with no cost barriers (aOR = 1.82 (95% CI, 1.54–2.15)). Having double cost barriers was associated with single CAM (aOR = 1.55 (95%, 1.21–2.00)) and ≥2 CAM use (aOR = 2.55 (95%, 1.95–3.34)) while the odds of having a triple cost barrier was 50% higher in one CAM users (aOR = 1.50 (95%, 1.09–2.05)) and 196% higher in ≥2 CAM users (aOR = 2.96 (95% CI, 2.17–4.01)) compared to non-CAM users. Single CAM users compared to non-CAM users had higher odds of not well controlled symptoms (aOR = 1.63 (95% CI, 1.46–1.82)) and very poorly controlled symptoms (aOR = 2.20 (95% CI, 1.92–2.51)). Adults who endorse ≥2 CAM use in comparison to non-CAM users were more likely to have very poorly controlled symptoms (aOR = 2.58 (95%, 2.22–3.00)) and not well controlled symptoms (aOR = 2.15 (95%, 1.88–2.45)) when compared to adults with well controlled symptoms.

## 4. Discussion

Few recent studies have examined the factors associated with CAM use among current US adults with asthma despite the increasing trends in the use of CAM. In our study, more than 45% of adults with current asthma reported ever using CAM, while about 25% and 20% reported using one CAM and ≥2 CAM, respectively. Our finding is consistent with other studies that reported the prevalence of CAM use among the asthma population [18,28,30,33]. Consistent with Andersen’s Behavioral Model of Healthcare Service Use, we showed that using CAM is associated with sociodemographic and health-related factors.

Males were less likely to ever use CAM, use one CAM, or use ≥2 CAM in our study compared to women. Our study agrees with other population-based studies that found women are more likely than men to use CAM [3,5,25,26,27,43]. Shaw et al. (2008) reported a 61% increased odds of CAM use among females compared to males [3]. The reason for this consistent association remains to be determined. However, some theories to explain this association include women being more open to the utilization of health services and the increased health-seeking behavior of females compared to males [44]. Some researchers have suggested modern society’s progressive and dynamic nature, which supports women’s independence and personal transformation through self-reflection and self-discovery [45].

Odds of CAM use were higher among multi-/other race/ethnicity compared to white non-Hispanics and were not significant when other races were compared to white non-Hispanics. Huo et al. (2015) found 60% and 50% increased odds of using CAM among “multiple” and “other” race/ethnicity compared to whites in their analysis of the 2010 ACBS, respectively [43], while Kim et al. (2020) noted a decreased odds of CAM use among Blacks and Hispanics compared to Whites [28]. Bishop et al. (2010) reported 38 studies showing that adults from ethnic minorities use CAM less than Whites and 15 studies that show the opposite [25], highlighting the complexity of this association. The reason for these differences needs to be clarified and may include the differences in the sample design, survey year used in analysis, and definitions of CAM and racial classifications that are used in these studies.

Middle-aged and older adults were less likely to use CAM than younger adults in our study. Shaw et al. (2008) reported an inverted U-shaped relationship with age and a 128% increase in odds among asthma patients 45–54 years compared to children aged 9–14 years [3]. Jourbert et al. (2012) reported the age group 35–51 years as more likely to endorse CAM use [23]. Marino and Shen (2010) did not find an association between age of CAM use in their analysis of the 2006 ACBS after adjusting for demographics and region [30]. The difference in the observed association may be due to differences in age categories used in other studies. Moreover, the proportion of CAM users among those aged ≤35 years in our study is higher than in the other studies. In addition, given that asthma is more common among younger than older adults, it is likely that younger people are more likely to use CAM for asthma; however, more studies to understand the effect of age on CAM use behavior are needed. 

Our results also show that there was a significant association between CAM use and one, two, and three cost barriers in a dose–response fashion. Furthermore, we found a dose–response association among users of single CAM and those using two or more CAM therapies and cost barriers. Interestingly, we observed a “lateral” dose–response relationship, where the odds ratio for individuals with ≥2 CAM use was higher than that for adults with single CAM use compared to non-users. Studies have suggested the high cost of healthcare and medications could be an incentive to use CAM instead of conventional treatment [13,18,30]. Pagan et al. (2005) found differences in CAM use across all CAM therapies when comparing the ability or inability of adults to obtain medical care because of cost after controlling for sociodemographic characteristics, income, and self-reported health status, implicating cost barrier as a factor for CAM use [13]. Among asthma patients, Bishop et al. (2010) showed that having a cost barrier in the last 12 months was associated with 180% increased odds of using CAM [25]. We also demonstrate a dose–response relationship between CAM use and multiple barriers, further providing evidence that as the number of cost barriers to asthma care increases, CAM use does not only increase but the number of CAM use increases as well. Our findings suggest that the use of CAM among asthma patients may reflect an increasing lack of access to asthma care for some patients and indicates that CAM may be used in an alternative rather than a complementary manner. CAM use in the US costs an estimated $34 billion per year in out-of-pocket expenditures [46]. Such out-of-pocket expenditures could be a testament to the belief that CAM/therapies have benefits that outweigh their costs and, as such, it is possible that asthma patients may try CAM first, thereby having less money available for conventional asthma treatment. Hence, CAM use could also be both an alternative and complementary therapy in that sense.

There was no significant association between income, insurance, and CAM use. Our finding on the relationship between income and complementary medicine use is consistent with one previous study that used a large population dataset [30] but differs from other studies that found CAM use to be linked to higher income [17,47,48] and lower income [41,49]. It is plausible that having insurance may increase CAM use, as insurance companies are increasingly covering CAM procedures, particularly office-based ones [48]. The National Center for Complementary and Integrative Health reports that insurance coverage for acupuncture (32% of large insurance firms), homeopathy (11% of insurance firms), and naturopathy (insurance more likely to cover a licensed naturopath) is increasing [50]. However, data also suggest that out-of-pocket costs for CAM are rising [46], which may indicate that other factors besides insurance are driving CAM use behavior. This discrepancy is noteworthy and warrants further investigation.

The presence of two or more comorbidities was identified as a significant predictor of CAM use. Andersen and colleagues (1995) have previously posited that health service utilization can be explained by several key variables, including physical and psychological symptoms, disease burden, and overall health status [34]. In the context of asthma, adults experiencing increased chronic disease burden and asthma symptoms that are unresponsive to conventional therapies may be more likely to seek out alternative treatments in the hopes of finding a cure [25]. Furthermore, our findings indicate that CAM use is associated with poorly controlled asthma, consistent with prior studies that have reported an association between poor asthma control and CAM use [16,18,29,30,31,32,33]. This association followed a dose–response relationship and remained significant after adjusting for all covariates in both binary and multinomial models. Studies have suggested that those with poorly controlled asthma are more likely to be dissatisfied with the conventional treatments and are more likely to be motivated to use CAM [16,18].

The present study boasts notable strengths. Firstly, the use of data from the ACBS allowed for the collection of information on alternative therapies from a nationally representative sample of non-institutionalized US adults, affording a highly accurate estimation of CAM use across diverse population subgroups. Additionally, the large sample size enabled comprehensive investigation of the factors associated with CAM use and other self-reported health characteristics, including symptoms of asthma control and income. The utilization of ACBS data also afforded the opportunity to scrutinize individual CAM therapies, which is of paramount importance given that CAM use represents a highly heterogeneous behavior characterized by varied effectiveness, intensity, and side effects. Combining all CAM modalities into a single cluster would result in the loss of the direction of effect of specific therapies. Therefore, the ACBS, with its categorization of CAM modalities into eleven different kinds of CAMs, effectively accounts for this heterogeneity.

Several limitations should be acknowledged in this study. Firstly, the use of cross-sectional data from the BRFSS precludes the determination of temporality between asthma severity and the use of alternative therapies. Additionally, selection bias may have been introduced as the BRFSS excludes institutionalized and hospitalized individuals, which could lead to an underestimation of the prevalence of active and severe asthma among adults. Such bias may also result in an overrepresentation of healthier individuals and those with greater access to alternative therapies, which could potentially bias the results towards the null. Furthermore, self-reported measures of asthma symptom status and CAM use may be susceptible to recall bias and misclassification. Lastly, the ACBS did not collect information on the dose and frequency of CAM use, limiting the ability to assess the intensity of alternative therapy behaviors.

## 5. Conclusions

Our study is the largest study to report the prevalence of CAM use among current asthma adults having used eight survey year datasets. The trend in CAM use among current asthma patients is increasing and our study supports other studies that found sociodemographic and health-related factors implicated in CAM use among adults with asthma. With the inclusion of breathing exercise as a useful supplement to asthma pharmacotherapy for symptoms and quality of life improvement in the GINA guidelines and ongoing clinical trial efforts in complementary/alternative therapies across the US, it is expected that CAM use would further increase among patients with asthma. Therefore, physicians and primary care providers should inquire about CAM use in a non-judgmental manner and to try to understand drivers of such perception and behavior. Our study also has policy implications, suggesting that cost barriers to asthma care may be a driving factor behind the use of CAM by patients. Therefore, policymakers should prioritize efforts to reduce these barriers to ensure that all asthma patients have access to affordable, evidence-based treatment options.

## Figures and Tables

**Table 1:** Sociodemographic and health factors among US adults with current asthma: Behavioral Risk Factor Surveillance System Asthma Call-back Survey, 2012–2019.

Characteristics			Ever Use of CAM			
	Survey Respondents		No		Yes	
	No, ^a^	% ^b^ (95% CI ^b^)	No, ^a^	% ^b^ (95% CI ^b^)	No, ^a^	% ^b^ (95% CI ^b^)
**Total**	76,802		44,347	54.8 (53.9–55.8)	32,455	45.2 (44.2–46.1)
**Gender ^c^**						
Female	53,478	64.2 (63.3–65.1)	29,639	52.0 (50.9–53.1)	23,839	48.0 (46.8–49.1)
Male	23,324	35.8 (34.9–36.7)	14,708	59.9 (58.3–61.5)	8616	40.1 (38.5–41.7)
**Age ^c^**						
≤35 years	9448	31.9 (30.9–32.9)	4689	49.1 (47.0–51.3)	4759	50.9 (48.7–53.0)
36–50 years	13,196	22.9 (22.2–23.7)	7144	54.0 (52.2–55.9)	6052	46.0 (44.1–47.8)
51–65 years	28,379	27.9 (27.2–28.6)	16,063	56.1 (54.8–57.4)	12,316	43.9 (42.6–45.2)
>65 years	25,779	17.2 (16.7–17.7)	16,451	64.3 (63.0–65.7)	9328	35.7 (34.3–37.0)
**Race/ethnicity ^c^**						
White non-Hispanic	60,295	68.2 (67.3–69.2)	35,642	56.8 (55.8–57.8)	24,653	43.2 (42.2–44.2)
Black non-Hispanic	4859	11.5 (10.8–12.2)	2736	52.8 (49.6–56.1)	2123	47.2 (43.9–50.4)
Multiple/Other race non-Hispanic	5209	6.7 (6.3–7.2)	2553	48.1 (44.4–51.8)	2656	51.9 (48.2–55.6)
Hispanic	5656	13.5 (12.7–14.3)	3036	51.0 (47.6–54.3)	2620	49.0 (45.7–52.4)
**Household income ^c^**						
>$75,000	17,348	29.7 (28.8–30.6)	10,942	60.0 (58.2–61.7)	6406	40.0 (38.3–41.8)
$50,000–$75,000	9779	13.3 (12.7–13.9)	5879	58.6 (56.2–61.1)	3900	41.4 (38.9–43.8)
$25,000–$49,999	16,005	21.4 (20.6–22.2)	9173	53.0 (50.9–55.0)	6832	47.0 (45.0–49.1)
<$25,000	24,657	35.6 (34.7–36.5)	13,082	51.1 (49.4–52.8)	11,575	48.9 (47.2–50.6)
**Health insurance ^c^**						
None	4646	10.2 (9.5–10.9)	2171	46.7 (42.9–50.6)	2475	53.3 (49.4–57.1)
Yes	72,013	89.7 (89.1–90.5)	42,100	55.8 (40.9–69.7)	29,913	44.2 (43.3–45.1)
**Cost Barrier ^c^**						
None	63,793	80.6 (79.9–81.4)	38,898	58.8 (57.8–59.8)	24,895	41.2 (40.2–42.2)
One cost barrier	8066	10.7 (10.1–11.2)	3740	42.8 (40.2–45.4)	4326	57.2 (54.6–59.8)
Two cost barriers	3049	5.4 (4.9–5.8)	1086	34.5 (30.1–38.9)	1963	65.5 (61.1–69.9)
Three cost barriers	1875	3.3 (3.0–3.7)	611	30.3 (25.4–35.2)	1264	69.7 (64.8–74.6)
**Comorbidity ^c^**						
None	10,776	21.2 (20.4–22.0)	6771	58.7 (56.4–61.0)	4005	41.3 (39.0–43.6)
One comorbidity	15,875	24.8 (24.0–25.7)	9570	56.3 (54.3–58.3)	6305	43.7 (41.7–45.7)
Two comorbidities	16,449	20.7 (19.9–21.4)	9630	54.3 (52.3–56.2)	6819	45.7 (43.8–47.7)
Three comorbidities	13,741	14.9 (14.3–15.5)	7769	53.0 (50.8–55.1)	5972	47.0 (44.9–49.2)
≥Four comorbidities	19,961	18.4 (17.8–19.0)	10,607	50.5 (48.9–52.2)	9354	49.5 (47.8–51.1)
**Asthma control ^c^**						
Very poorly controlled	19,134	22.4 (21.6–23.1)	9126	42.5 (40.8–44.3)	10,008	57.5 (55.7–59.2)
Not well controlled	27,663	36.0 (35.1–36.9)	14,503	49.5 (47.9–51.1)	13,160	50.5 (48.9–52.1)
Well controlled	30,005	41.6 (40.7–42.5)	20,718	66.0 (64.7–67.4)	9287	34.0 (32.6–35.3)

CI, confidence interval; CAM, complementary and alternative medicine. ^a^ Unweighted pooled sample size, 2012–2019. Due to item non-response, individual characteristic categories may not sum to total. ^b^ Weighted prevalence and 95% confidence interval. ^c^ *p* Values < 0.05 for the chi-square test of association between ever use of CAM and all selected variables.

**Table 2:** Sociodemographic and health factors among US adults with current asthma: Behavioral Risk Factor Surveillance System Asthma Call-back Survey, 2012–2019.

Characteristics			Number of CAM Use					
	Survey Respondents		No CAM Use		One CAM Use		≥Two CAM Use	
	No, ^a^	% ^b^ (95% CI ^b^)	No, ^a^	% ^b^ (95% CI ^b^)	No, ^a^	% ^b^ (95% CI ^b^)	No, ^a^	% ^b^ (95% CI ^b^)
**Total**	76,802		44,347	54.8 (53.9–55.8)	18,737	25.3 (24.5–26.1)	13,718	19.9 (19.1–20.6)
**Gender ^c^**								
Female	53,478	64.2 (63.3–65.1)	29,639	52.0 (50.9–53.1)	13,448	26.3 (25.3–27.3)	10,391	21.7 (20.8–22.7)
Male	23,324	35.8 (34.9–36.7)	14,708	59.9 (58.3–61.5)	5289	23.6 (22.2–24.9)	3327	16.5 (15.2–17.8)
**Age ^c^**								
≤35 years	9448	31.9 (30.9–32.9)	4689	49.1 (47.0–51.3)	2620	27.8 (25.9–29.6)	2139	23.1 (21.3–24.9)
36–50 years	13,196	22.9 (22.2–23.7)	7144	54.0 (52.2–55.9)	3277	25.1 (23.4–26.8)	2775	20.8 (19.3–22.3)
51–65 years	28,379	27.9 (27.2–28.6)	16,063	56.1 (54.8–57.4)	6949	24.6 (23.4–25.7)	5367	19.3 (18.3–20.3)
>65 years	25,779	17.2 (16.7–17.7)	16,451	64.3 (63.0–65.7)	5891	22.1 (21.0–23.2)	3437	13.6 (12.5–14.6)
**Race/ethnicity ^c^**								
White non-Hispanic	60,295	68.2 (67.3–69.2)	35,642	56.8 (55.8–57.8)	14,556	24.6 (23.8–25.6)	10,097	18.6 (17.8–19.4)
Black non-Hispanic	4859	11.5 (10.8–12.2)	2736	52.8 (49.6–56.1)	1293	28.6 (25.6–31.6)	830	18.6 (16.2–20.9)
Multiple/Other race non-Hispanic	5209	6.7 (6.3–7.2)	2553	48.1 (44.4–51.8)	1328	27.7 (24.2–31.2)	1328	24.3 (21.3–27.2)
Hispanic	5656	13.5 (12.7–14.3)	3036	51.0 (47.6–54.3)	1346	25.0 (22.2–27.9)	1274	24.0 (21.2–26.8)
**Household income ^c^**								
>$75,000	17,348	29.7 (28.8–30.6)	10,942	60.0 (58.2–61.7)	3719	22.6 (25.9–24.1)	2687	17.4 (16.0–18.8)
$50,000–$75,000	9779	13.3 (12.7–13.9)	5879	58.6 (56.2–61.1)	2266	23.7 (21.6–25.8)	1634	17.7 (15.6–19.7)
$25,000–$49,999	16,005	21.4 (20.6–22.2)	9173	53.0 (50.9–55.0)	3868	25.4 (23.6–27.1)	2964	21.7 (20.0–23.4)
<$25,000	24,657	35.6 (34.7–36.5)	13,082	51.1 (49.4–52.8)	6654	27.1 (25.7–28.6)	4921	21.7 (20.4–23.1)
**Health insurance ^c^**								
None	4646	10.2 (9.5–10.9)	2171	46.7 (42.9–50.6)	1317	28.2 (25.1–31.3)	1158	25.1 (22.0–28.1)
Yes	72,013	89.7 (89.1–90.5)	42,100	55.8 (40.9–69.7)	17,389	25.0 (24.2–25.8)	12,524	19.2 (18.4–20.0)
**Cost Barrier ^c^**								
None	63,793	80.6 (79.9–81.4)	38,898	58.8 (57.8–59.8)	14,992	24.3 (23.4–25.2)	9903	16.9 (16.2–17.7)
One cost barrier	8066	10.7 (10.1–11.2)	3740	42.8 (40.2–45.4)	2318	29.1 (26.6–31.5)	2008	28.1 (25.5–30.7)
Two cost barriers	3049	5.4 (4.9–5.8)	1086	34.5 (30.1–38.9)	894	30.8 (27.0–34.6)	1069	34.7 (30.6–38.7)
Three cost barriers	1875	3.3 (3.0–3.7)	611	30.3 (25.4–35.2)	527	29.2 (24.4–34.0)	737	40.4 (35.4–45.5)
**Comorbidity ^c^**								
None	10,776	21.2 (20.4–22.0)	6771	58.7 (56.4–61.0)	2325	23.1 (21.1–25.1)	1680	18.2 (16.2–20.1)
One comorbidity	15,875	24.8 (24.0–25.7)	9570	56.3 (54.3–58.3)	3628	24.9 (23.2–26.5)	2677	18.8 (17.3–20.3)
Two comorbidities	16,449	20.7 (19.9–21.4)	9630	54.3 (52.3–56.2)	3924	25.6 (23.9–27.4)	2895	20.1 (18.5–21.8)
Three comorbidities	13,741	14.9 (14.3–15.5)	7769	53.0 (50.8–55.1)	3409	26.3 (24.3–28.3)	2563	20.8 (19.1–22.5)
≥Four comorbidities	19,961	18.4 (17.8–19.0)	10,607	50.5 (48.9–52.2)	5451	27.3 (25.7–28.8)	3903	22.2 (20.7–23.7)
**Asthma control ^c^**								
Very poorly controlled	19,134	22.4 (21.6–23.1)	9126	42.5 (40.8–44.3)	5509	31.5 (29.7–33.3)	4499	26.0 (24.3–27.6)
Not well controlled	27,663	36.0 (35.1–36.9)	14,503	49.5 (47.9–51.1)	7395	26.7 (25.4–28.0)	5765	23.7 (22.3–25.1)
Well controlled	30,005	41.6 (40.7–42.5)	20,718	66.0 (64.7–67.4)	5833	20.7 (19.5–21.9)	3454	13.2 (12.2–14.2)

CI, confidence interval; CAM, complementary and alternative medicine. ^a^ Unweighted pooled sample size, 2012–2019. Due to item non-response, individual characteristic categories may not sum to total. ^b^ Weighted prevalence and 95% confidence interval. ^c^ *p* Values < 0.05 for the chi-square test of association between ever use of CAM and all selected variables.

**Table 3:** Multivariable logistic regression of sociodemographic and health factors associated with CAM use among US adults with current asthma: Behavioral Risk Factor Surveillance System Asthma Call-back Survey, 2012–2019.

Variables	Unadjusted OR (95% CI)	Adjusted OR (95% CI) ^a^
**Gender**		
Female	Ref	Ref
Male	0.73 (0.67–0.79)	0.7 (0.64–0.76)
**Age**		
≤35 years	Ref	Ref
36–50 years	0.82 (0.73–0.92)	0.71 (0.62–0.81)
51–65 years	0.76 (0.68–0.84)	0.6 (0.54–0.68)
>65 years	0.54 (0.48–0.60)	0.43 (0.38–0.49)
**Race/ethnicity**		
White non-Hispanic	Ref	Ref
Black non-Hispanic	1.17 (1.02–1.35)	1.10 (0.93–1.27)
Multiple/Other race non-Hispanic	1.42 (1.22–1.66)	1.37 (1.16–1.63)
Hispanic	1.27 (1.10–1.45)	0.98 (0.84–1.15)
**Household income**		
>$75,000	Ref	Ref
$50,000–$75,000	1.06 (0.93–1.20)	0.94 (0.82–1.10)
$25,000–$49,999	1.33 (1.19–1.49)	1.04 (0.92–1.17)
<$25,000	1.43 (1.30–1.58)	0.93 (0.83–1.04)
**Health insurance**		
None	Ref	Ref
Yes	0.70 (0.59–0.81)	0.93 (0.78–1.14)
**Cost Barrier**		
None	Ref	Ref
One cost barrier	1.90 (1.70–2.13)	1.54 (1.36–1.76)
Two cost barriers	2.71 (2.22–2.31)	1.97 (1.56–2.48)
Three cost barriers	3.28 (2.59–4.15)	2.10 (1.60–2.75)
**Comorbidity**		
None	Ref	Ref
One comorbidity	1.11 (0.98–1.25)	1.09 (0.94–1.25)
Two comorbidities	1.20 (1.06–1.36)	1.17 (1.02–1.35)
Three comorbidities	1.26 (1.11–1.44)	1.28 (1.09–1.51)
≥Four comorbidities	1.39 (1.24–1.57)	1.42 (1.22–1.66)
**Asthma control**		
Well controlled	Ref	Ref
Not well controlled	1.98 (1.81–2.17)	1.83 (1.66–2.02)
Very poorly controlled	2.63 (2.39–2.89)	2.34 (2.09–2.62)

CI, confidence interval; OR, Odds Ratio; CAM, complementary and alternative medicine. ^a^ Adjusted for the variables listed in this table, smoking, and survey year.

**Table 4:** Multinomial logistic regression of sociodemographic and health factors associated with CAM use among US adults with current asthma: Behavioral Risk Factor Surveillance System Asthma Call-back Survey, 2012–2019.

Variables	One CAM Use ^a^	≥Two CAM Use ^a^
	Adjusted OR (95% CI) ^b^	Adjusted OR (95% CI) ^b^
**Gender**		
Female	Ref	Ref
Male	0.74 (0.67–0.82)	0.65 (0.57–0.73)
**Age**		
≤35 years	Ref	Ref
36–50 years	0.72 (0.62–0.83)	0.69 (0.58–0.82)
51–65 years	0.64 (0.56–0.74)	0.56 (0.48–0.66)
>65 years	0.50 (0.43–0.58)	0.35 (0.30–0.42)
**Race/ethnicity**		
White non-Hispanic	Ref	Ref
Black non-Hispanic	1.16 (0.97–1.38)	0.98 (0.81–1.19)
Multiple/Other race non-Hispanic	1.28 (1.03–1.58)	1.51 (1.24–1.83)
Hispanic	0.88 (0.73–1.05)	1.13 (0.93–1.37)
**Household income**		
>$75,000	Ref	Ref
$50,000–$75,000	0.98 (0.84–1.14)	0.89 (0.74–1.08)
$25,000–$49,999	1.05 (0.92–1.21)	1.02 (0.87–1.20)
<$25,000	1.00 (0.87–1.14)	0.84 (0.73–0.98)
**Health insurance**		
None	Ref	Ref
Yes	0.88 (0.72–1.07)	1.00 (0.80–1.26)
**Cost Barrier**		
None	Ref	Ref
One cost barrier	1.36 (1.17–1.57)	1.82 (1.54–2.15)
Two cost barriers	1.55 (1.21–2.00)	2.55 (1.95–3.34)
Three cost barriers	1.50 (1.09–2.05)	2.96 (2.17–4.01)
**Comorbidity**		
None	Ref	Ref
One comorbidity	1.07 (0.91–1.25)	1.11 (0.92–1.35)
Two comorbidities	1.14 (0.97–1.34)	1.21 (1.01–1.47)
Three comorbidities	1.22 (1.01–1.47)	1.38 (1.12–1.70)
≥Four comorbidities	1.32 (1.11–1.57)	1.58 (1.29–1.94)
**Asthma control**		
Well controlled	Ref	Ref
Not well controlled	1.63 (1.46–1.82)	2.15 (1.88–2.45)
Very poorly controlled	2.20 (1.92–2.51)	2.58 (2.22–3.00)

CI, confidence interval; OR, odds ratio; CAM, complementary and alternative medicine. ^a^ No CAM use is the comparator. ^b^ Adjusted for the variables listed in this table, smoking, and survey year.

## Data Availability

The data used to generate the findings of this study are publicly available in the CDC Asthma Call-Back Survey Website available at: https://www.cdc.gov/brfss/acbs/index.htm (accessed on 1 January 2023). The Asthma Call-back Survey (ACBS) is a product of CDC’s National Asthma Control Program (NACP).
